# An active Helitron transposon family in wheat

**DOI:** 10.1038/s41477-026-02319-3

**Published:** 2026-06-05

**Authors:** Haoran Peng, Lijing Tang, Nataliya Hrunyk, Roman Kellenberger, Dario Fossati, Hélène Rimbert, Pierre Sourdille, Shengwei Ma, Alison B. Hickman, Fred Dyda, Claudia Köhler, Carole Charlier, Frédéric Choulet, Etienne Bucher

**Affiliations:** 1 Crop Genome Dynamics Group, Agroscope, Nyon, Switzerland; 2 Department of Plant Reproductive Biology and Epigenetics, Max Planck Institute of Molecular Plant Physiology, Potsdam, Germany; 3 Unit of Animal Genomics, GIGA & Faculty of Veterinary Medicine, University of Liège, Liège, Belgium; 4 Field Crop Breeding and Genetic Resources, Agroscope, Nyon, Switzerland; 5 INRAE, GDEC, Université Clermont Auvergne, Clermont-Ferrand, France; 6 Yazhouwan National Laboratory, Sanya, China; 7 Laboratory of Molecular Biology, National Institute of Diabetes and Digestive and Kidney Diseases, National Institutes of Health, Bethesda, MD USA

**Keywords:** Plant genetics, Mobile elements

## Abstract

Transposable elements play a pivotal role in genome evolution and phenotypic variation in numerous eukaryotic species^1^. Helitrons, a recently identified category of transposons, remain poorly understood in terms of epigenetic regulation and real-time mobilization in plants^2,3^. Here our study reveals that reduced DNA methylation combined with heat stress promotes the mobilization of the *Xuan*–*Feng* Helitron family in wheat. Activation is marked by transcription, extrachromosomal circular DNA formation and novel somatic insertions. Genetic segregation and heterologous reconstitution establish *Feng8* as the autonomous driver of the *Xuan*–*Feng* family mobilization. These findings represent a step forward in the study of active Helitrons and their potential biological functions as well as their role in genome dynamics and their potential use in crop breeding.

## Abstract (web summary)

Helitrons are a recently identified category of transposons. This study reveals that heat stress combined with reduced DNA methylation can mobilize a Helitron family in wheat. Its mobilization is genetically linked to a specific full-length member catalysing the full Helitron life cycle.

## Main

The genomes of most plant species predominantly consist of transposable elements (TEs)^4^. Hexaploid bread wheat (*Triticum aestivum*), the most important crop worldwide, exhibits a large genome size (16 Gb), with more than 85% originating from TEs and other repetitive sequences^5^. The significance of TEs in the functional and evolutionary dynamics of genomes is now widely acknowledged. TEs contribute to the regulation of gene expression, stress responses and adaptation, and they played key roles during crop domestication^6–9^. Helitrons, a distinct category of DNA TEs, eluded discovery until the early 2000s when bioinformatic methodologies enabled their identification over 50 years after the initial discovery of TEs^2,10^. Helitrons have been suggested to employ a rolling-circle transposition mode reminiscent of plasmid replication and single-stranded DNA viruses^11^. This transposition mode is distinct, as it does not generate target site duplications, a characteristic footprint of many TEs^12^.

**Fig. 1:**
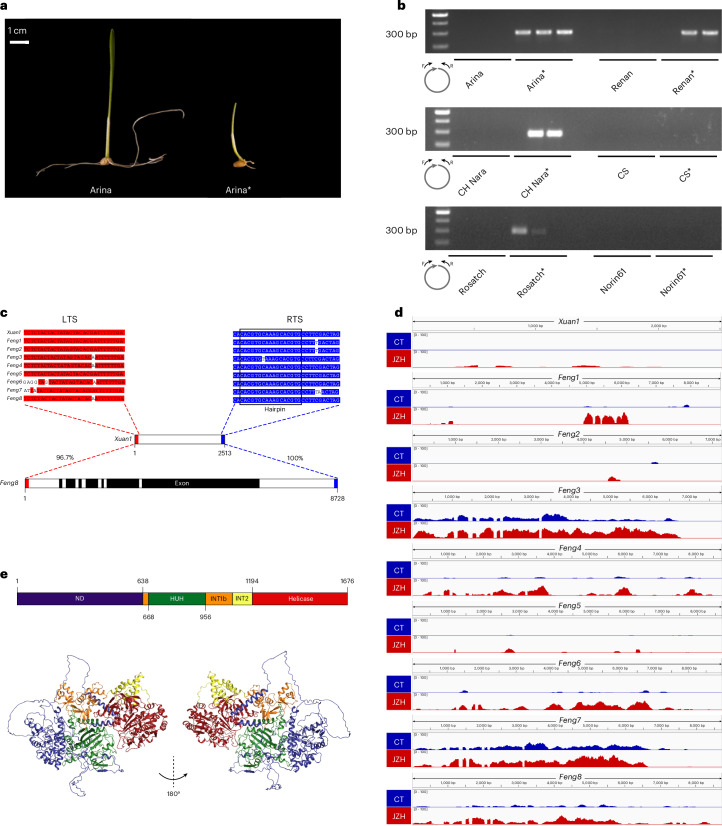
Detection of Helitron-derived eccDNA and characterization of the *Xuan*–*Feng* Helitron family in wheat. **a**, Morphological comparison of a seven-day Arina control seedling (left) and a JZH-treated seedling (right). **b**, Detection of Helitron eccDNA using inverse PCR on RCA-treated DNA as a template. Primer localizations are depicted on the left (F and R). The first lane shows the GeneRuler 100-bp DNA ladder; each cultivar was tested with three biological replicates issued from the control and JZH-treated groups. The asterisks indicate JZH-treated samples. **c**, Alignments of the LTS (red) and RTS (blue) of *Xuan1* and the eight *Feng* members. The consensus bases are highlighted. **d**, Genome browser views showing the normalized transcript levels assessed by RNA-seq for *Xuan1* and the eight *Feng* sequences under control conditions (blue) and under JZH treatment (red). One out of three biological replicates is shown here (additional replicates are shown in Extended Data Fig. 4) **e**, Domains of the FENG8 transposase and two views of the predicted 3D structure (rotated by 180°).

**Extended Data Fig. 1:**
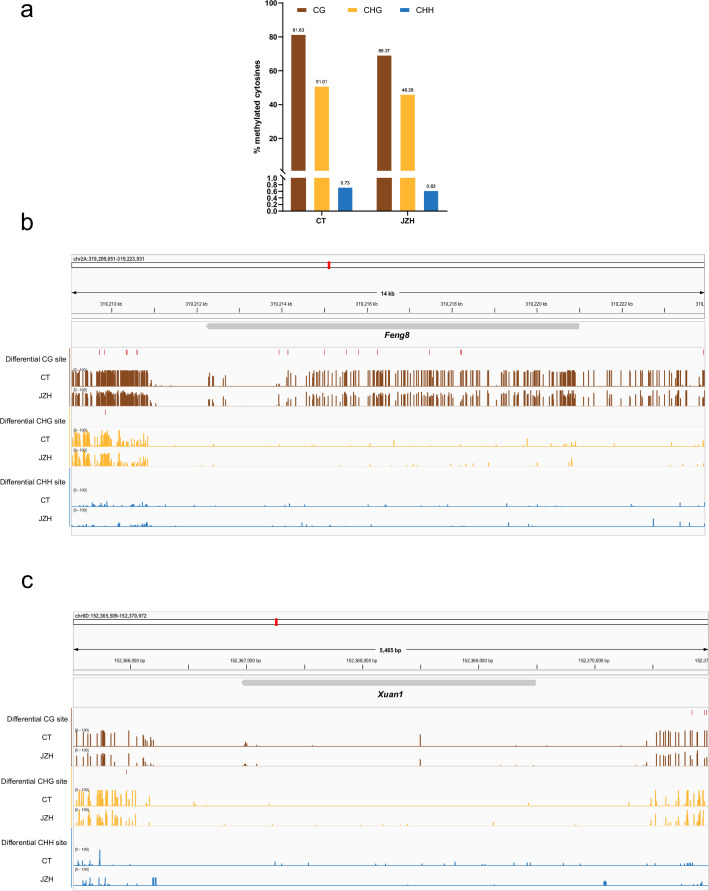
Genome-wide and locus-specific DNA methylation changes following JZH treatment. **a**, Global levels of cytosine methylation across the three sequence contexts (CG, CHG, and CHH) in untreated and JZH-treated plants. **b**, Integrative Genomics Viewer (IGV) visualization of cytosine methylation in the *Feng8* locus. Tracks represent CG (brown), CHG (yellow), and CHH (blue) methylation. Significantly differentially methylated cytosine positions are highlighted in red. **c**, IGV visualization of cytosine methylation in the *Xuan1* locus.

**Extended Data Fig. 2:**
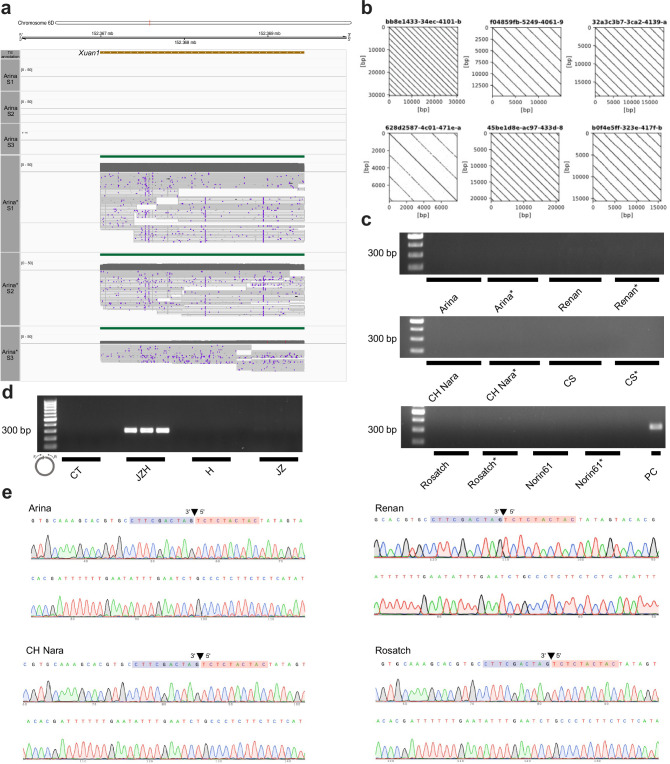
Identification and verification of *Helitron* eccDNA. **a**, Nanopore eccDNA sequencing analysis of untreated and treated Arina seedlings. The chromosomal position is shown on the top, the first row presents our *Helitron* annotation of the Arina*LrFor* genome^31^. Each row below the TE annotation represents an individual sample with three biological replicates from the control and JZH treated wheat seedlings. The green lines depict the eccDNA loci that were detected by ecc_finder^29^, the dark gray oblong shape indicates normalized read coverage and below are the individual Nanopore reads mapped to this region (gray reads are single-mapped and white reads mapped to multiple loci in the genome). **b**, Dot-plot analyses visualizing the self-similarity of selected single long reads containing tandem repeats of the *Helitron* in JZH-treated Arina. **c**, Confirmation of absence of genomic *Xuan1* tandem repeats in the corresponding wheat varieties using inverse PCR with genomic DNA as template (no RCA), the primer localization depicted on the left (F, R). The first lane shows the GeneRuler 100 bp DNA ladder, each cultivar was tested using three biological replicates issued from the control and JZH-treated groups. RCA eccDNA from treated Arina plants was used as a template for the positive controls (PC). *Xuan1* eccDNA can only be detected following RCA. The asterisk (*) indicates JZH-treated samples. **d**, Detection of *Helitron* eccDNA in the Arina variety using inverse PCR on RCA-amplified eccDNA; the primer localization depicted on the left (F, R). The first lane shows the GeneRuler 100 bp DNA ladder; each treatment was tested with three biological replicates issued from the control (CT), JZH treatment, heat treatment (H), and JZ-treatment groups. **e**, Chromatograms from Sanger sequencing, the blue background highlights the 10 bp of *Helitron* RTS, and the red background highlights the 10 bp of *Helitron* LTS, and the inverted black triangle indicates the circle junction.

A canonical Helitron has conserved termini: a 5′ TC, a 3′ CTRR sequence (where R stands for A or G) and a 16–20-bp hairpin structure located 10–15 bp from the 3′ end^2,13^. Due to the high diversity of their internal sequences, Helitron families are defined according to sequences of 30 bases that are present in the termini and referred to as the left terminal sequence (LTS) and the right terminal sequence (RTS)^14,15^. Whereas their relics are abundant across the eukaryotic kingdom and indicative of historical activity^16^, direct evidence of real-time Helitron mobility is scarce^17^. Indeed, controllable active TE mobility is rarely observed in plants, largely due to the concerted action of multiple epigenetic mechanisms that silence them^18,19^; for example, DNA methylation and small RNA pathways robustly suppress their mobility^1,20^. Given that Helitrons employ extrachromosomal circular DNA (eccDNA) intermediates during their transposition process^21,22^, these eccDNAs may serve as valuable diagnostic markers for assessing Helitron activity. To promote TE mobilization using a similar approach as previously reported^23^, we treated wheat seedlings with a combination of two chemicals that perturb transcriptional and epigenetic homeostasis: the RNA polymerase I, II and III inhibitor juglone (J)^24,25^ and the DNA methylation inhibitor zebularine (Z)^26,27^. Since numerous TEs are stress-responsive^28^, we combined the drug treatments (JZ) with heat stress (H). In the wheat cultivar Arina, the JZH-treated seedlings exhibited substantially reduced vigour, characterized by shorter roots and delayed leaf development (Fig. 1a). Nanopore-based whole-genome sequencing and DNA methylation calling revealed that global DNA methylation across the three sequence contexts (CG, CHG and CHH) was reduced following JZH treatment. In the CG, CHG and CHH methylation contexts, the levels decreased by 15%, 9.3% and 14%, respectively, compared with control plants (Extended Data Fig. 1a). To identify activated Helitrons, eccDNAs from these plants were extracted, amplified by rolling circle amplification (RCA) and subjected to long-read sequencing^29,30^. We observed that JZH-treated samples generated a unique eccDNA, precisely overlapping with a Helitron TE annotation (2,513 bp, chromosome 6D: 152366979–152369491 of the Arina*LrFor* reference genome^31^), whereas this signal was absent in the control samples (Extended Data Fig. 2a). Dot-plot graphs generated from the long reads revealed numerous reads containing multiple tandem repeat copies of the Helitron sequence. These repeats were the result of the in vitro RCA reaction that was used to amplify the eccDNA signal (Extended Data Fig. 2b), thus confirming the circular nature of the original DNA templates.

Next, to independently confirm the presence of Helitron eccDNA, we conducted inverse PCR using primers designed to span the eccDNA junction (Fig. 1b). We ruled out any non-specific signals potentially originating from genomic tandem repeat sequences in the Arina genome, as we did not detect any PCR products when genomic DNA was used as a PCR template (Extended Data Fig. 2c). To determine the requirements for efficient Helitron eccDNA production, we subjected plants to individual treatments with drugs alone (JZ), heat stress alone (H) and their combination (JZH). Helitron-derived eccDNA was exclusively detected via inverse PCR on RCA-amplified DNA from the JZH group (Extended Data Fig. 2d). These results demonstrate that Helitron-derived eccDNA accumulation is heat stress responsive but repressed by DNA methylation and silencing.

We then wanted to test whether eccDNA accumulation varied between distantly related wheat cultivars. We tested four European cultivars (Arina, Renan, CH Nara and Rosatch) and two Asian cultivars (Chinese Spring (CS) and Norin61). JZH-treated Arina, Renan, CH Nara and Rosatch produced detectable Helitron-derived eccDNAs, but the untreated control groups did not (Fig. 1b). Conversely, the cultivars CS and Norin61 did not produce detectable levels of Helitron-derived eccDNAs following JZH treatment (Fig. 1b). This demonstrates that not all wheat varieties have the capacity to activate this element even though it is present in their genome. To delineate the exact junction between the RTS and LTS of this Helitron in the varieties that showed eccDNA accumulation, we subjected the obtained PCR fragments to Sanger sequencing. We found that the junctions presented the exact expected RTS and LTS junctions in these varieties (Extended Data Fig. 2e).

**Extended Data Fig. 3:**
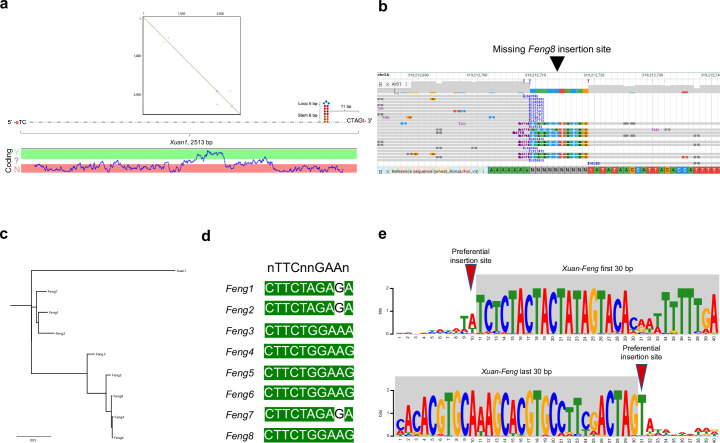
Sequence features of the *Xuan–Feng Helitron* family. **a**, Self-dot plot of the *Xuan1* sequence and schematic representation of the molecular features. The graph below indicates the protein coding capacity of the sequence, with red indicating low and green indicating high. The hairpin structure is depicted as colored rings. **b**, Genome browser view of the *Feng8* locus on chr2A:319,212,266 of the Arina*LrFor* reference genome where the *de novo* assembly failed to assemble *Feng8* (as indicated by the multiple N’s in the reference sequence shown on the bottom). The gray horizontal bars represent individual nanopore sequencing reads and insertions (numbers with magenta backgrounds) and mismatches (colored bases) compared to the reference sequence. **c**, Phylogenetic tree depicting the relationship between *Feng* members, *Xuan1* was included as an outlier. **d**, Comparison of the predicted heat response element (HRE) motif in the eight *Feng* members. The consensus bases are highlighted. **e**, Sequence motif logo illustrating the 40 bp boundaries of the *Xuan–Feng* family members, including 10 bp upstream and downstream sequences for each 5′ and 3′ ends. The gray background highlights the *Helitron* sequence.

The eccDNA-producing Helitron exhibited canonical features, initiating with a TC dinucleotide and terminating with a CTAG tetranucleotide (Fig. 1c). We also identified a 17-nucleotide hairpin structure, including a 5-nucleotide loop and a 6-nucleotide stem, located 11 nucleotides upstream of the RTS (Extended Data Fig. 3a). On the basis of the absence of transposase coding capability (Extended Data Fig. 3a), we concluded that this element was a non-autonomous Helitron and designated it *Xuan1*. Non-autonomous TEs do not encode a functional transposase necessary for self-transposition. Instead, they rely on *trans*-mobilization facilitated by the enzymatic machinery of their autonomous counterparts from the same family^12^. To delineate this Helitron family, we employed a classification approach based on the 30- bp LTS and RTS regions^14,15^. We designated this family *Xuan*–*Feng* (meaning ‘whirlwind’ in Chinese; *Xuan* signifies non-autonomous family members, and *Feng* denotes potentially autonomous ones). The annotation of this family identified *Xuan1–77* and *Feng1–7* (Supplementary Table 1). In addition, by employing whole-genome sequencing with Nanopore reads, we discovered *Feng8*, which was not completely assembled in the Arina*LrFor* reference genome^31^ (Fig. 1c, Extended Data Fig. 3b and Supplementary Table 1).

The *Xuan1* element and putatively autonomous *Feng* members exhibited conserved LTS and RTS, except for *Feng6* and *Feng7*, which had accumulated mutations in their LTS (Fig. 1c). Using a multiple alignment approach, *Feng* members were clustered into two groups: one group that included *Feng1*, *Feng2* and *Feng7* and a second group that contained all other *Feng* copies. Notably, *Feng4*, *Feng6* and *Feng8* showed over 99% nucleotide sequence identity (Extended Data Fig. 3c). We also detected a predicted heat response element with the consensus sequence nTTCnnGAAn in the promoter regions of *Feng3–6* and *Feng8* (Extended Data Fig. 3d). Notably, the *Arabidopsis* retrotransposon *ONSEN* (*ATCOPIA78*) was found to include the same sequence motif in its heat-stress-responsive promotor^32^. We also observed a preference for insertions of this Helitron family at AT dinucleotides (Extended Data Fig. 3e), which is a characteristic of Helitrons observed across different species^11^.

**Extended Data Fig. 4:**
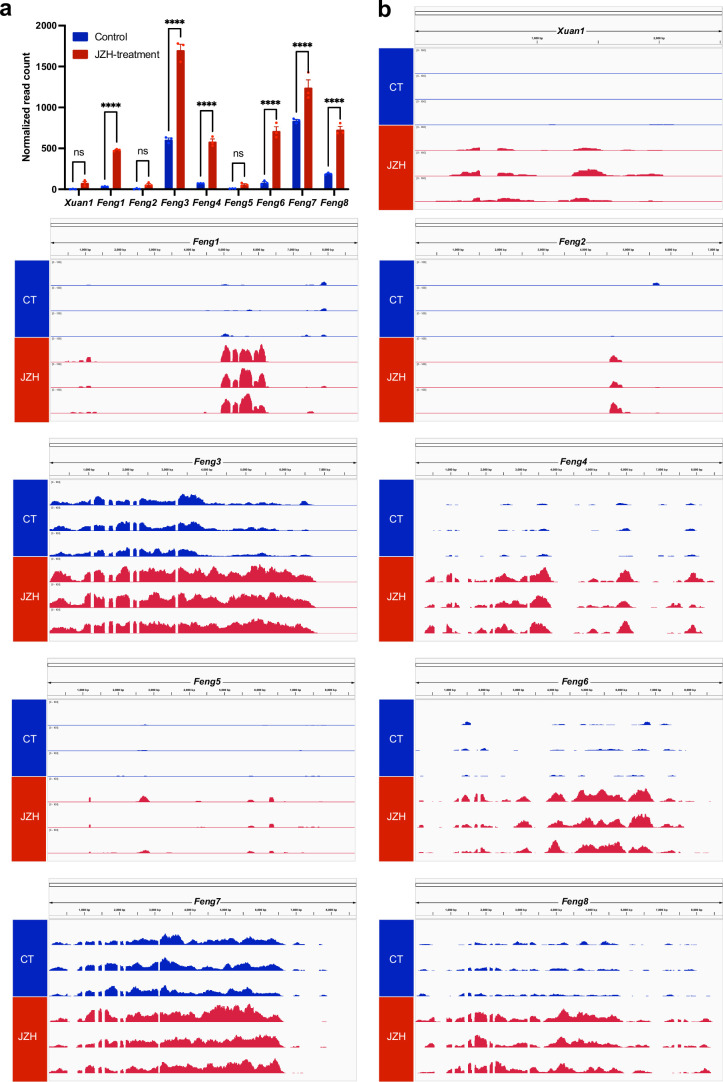
Transcript levels of *Xuan1* and *Feng1-8* assessed by RNA-seq. **a**, Quantification of the RNA-seq data obtained from control (blue) and JZH- treated (red) Arina seedlings. Three biological replicates derived from independent plants were analyzed. The statistics use two-way ANOVA of normalized read count mean with SEM; the stars indicate the adjusted *P* value < 0.0001. The exact *P* values are provided in Source Data Extended Data Fig. 4. **b**, Genome browser views showing three biological replicates of normalized transcript levels assessed by RNA-seq for *Xuan1* and the eight *Feng* sequences under control conditions (blue) and resulting from JZH treatment (red). Source data

**Extended Data Fig. 5:**
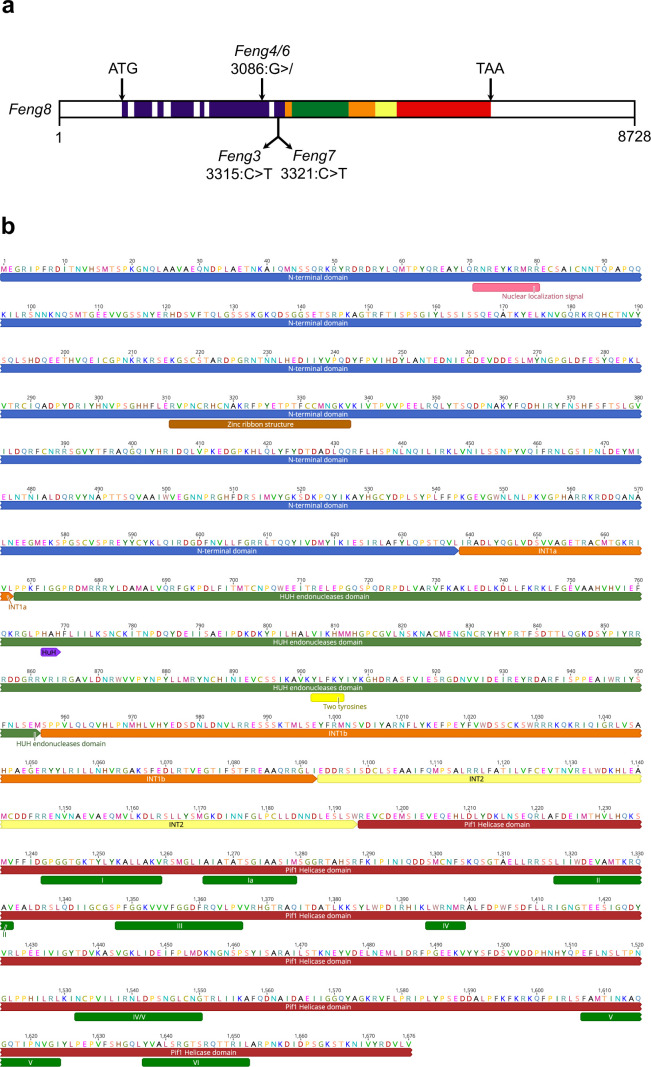
Graphical representation of the FENG8 protein sequence highlighting the conserved domains of the transposase. **a**, Schematic diagram depicting mutations that impact the protein coding sequences in *Feng3* (nonsense mutation), *Feng4* (frameshift mutation), *Feng6* (frameshift mutation), and *Feng7* (frameshift mutation) represented on the intact *Feng8* sequence. The colors on the exons correspond to the different transposase domains shown in Fig. 2. **b**, Residues involved in the zinc ribbon are highlighted in brown, the HUH motif is highlighted in purple, the Y2 motif in yellow, and the eight Helicase motifs are indicated in green.

To identify the autonomous *Feng* copy that facilitated the biosynthesis of *Xuan1* eccDNA, we examined the transcriptional activity of all *Feng* members using RNA sequencing (RNA-seq) to determine whether they responded to the JZH treatment. We observed only minimal, statistically non-significant, transcript levels for *Xuan1* in response to the JZH treatment (Fig. 1d). By contrast, the *Feng* elements displayed varying transcriptional responses following JZH treatment. *Feng2* and *Feng5* did not exhibit significant transcriptional activation, while *Feng1*, *Feng3*, *Feng4* and *Feng6* to *Feng8* showed significantly increased transcript levels upon JZH treatment (Fig. 1d and Extended Data Fig. 4). *Feng1* produced only a short 1,284-bp transcript following JZH treatment. Although *Feng3* and *Feng7* exhibited a partial constitutive transcriptional signal under control conditions, complete transcripts and significantly increased transcript levels were only observed in the JZH-treated group (Fig. 1d and Extended Data Fig. 4). A close inspection of the transcripts produced by *Feng3*, *Feng4*, *Feng6*, *Feng7* and *Feng8* revealed that all *Feng* copies, except *Feng8*, carried nonsense mutations or a frameshift mutation in the coding sequence (Extended Data Fig. 5a). We thus concluded that only *Feng8* had the capacity to code for a functional transposase. Next, we examined whether DNA methylation levels are affected at *Feng8* in JZH-treated plants. In untreated plants, the region was predominantly methylated in the CG context. Following JZH treatment, a subset of CG and CHG sites showed reduced methylation levels, consistent with increased transcriptional activity (Fig. 1d and Extended Data Fig. 1b). In contrast, the *Xuan1* locus exhibited low levels of DNA methylation across all sequence contexts in untreated plants, and this hypomethylated state was maintained after JZH treatment (Extended Data Fig. 1c).

On the basis of the RNA-seq data, we found that *Feng8* contained a coding sequence spanning 5,028 bp and encoded a transposase of 1,676 amino acids (Fig. 1e and Extended Data Fig. 5). This coding sequence consists of seven exons, and the first five introns start with a GT donor site and end with the AG acceptor site, whereas the last intron starts with GC. To verify the functional protein domains, we aligned FENG8 with the Helraiser protein (an in silico reconstituted Helitron transposase from *Myotis lucifugus*)^33^. We noticed that the FENG8 transposase had a larger N-terminal domain (ND) (Met1–Leu638) and harboured a potential zinc-finger-like motif (Arg311–Val337). The RepHel core of FENG8 comprised a HUH endonuclease domain spanning 289 amino acids (Pro668–Met956), featuring the conserved HUH motif (His767 and His769), as well as two tyrosine residues (Tyr902 and Tyr906) known to form the active site in Helraiser. Additionally, it encompassed a helicase domain spanning 483 (Arg1194–Val1676) amino acids, containing the eight conserved motifs that are characteristic of the SF1 superfamily of DNA helicases (Extended Data Fig. 5b).

**Extended Data Fig. 6:**
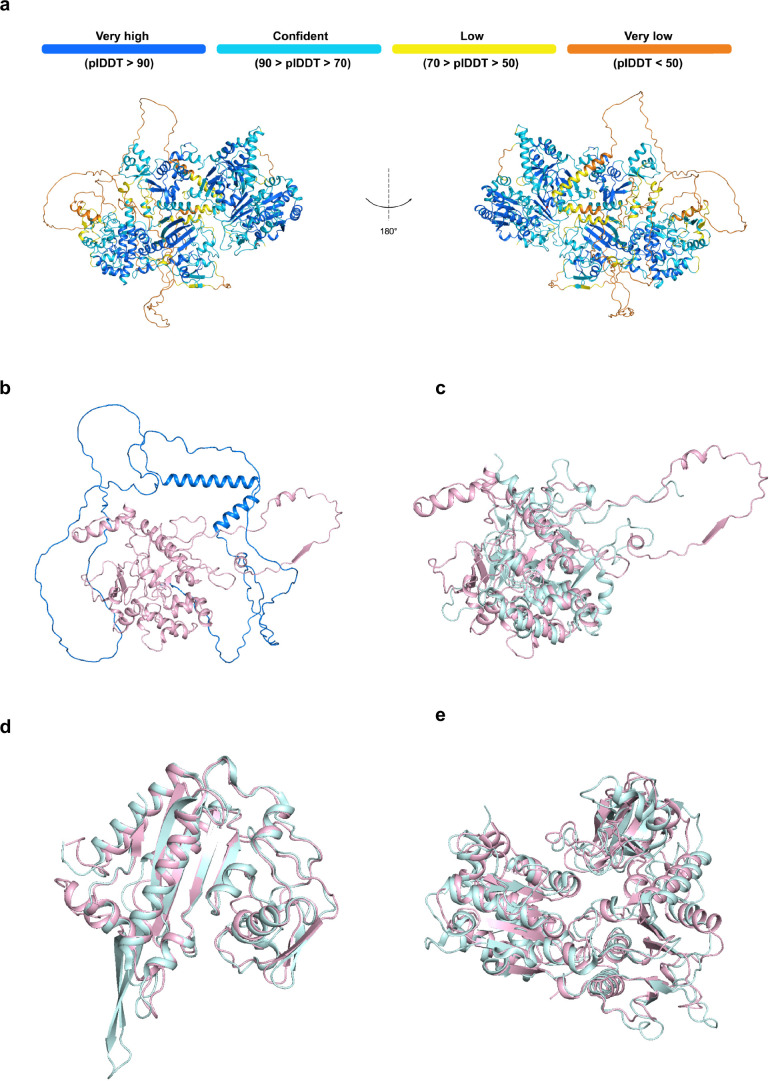
Structural comparison of FENG8 and Helraiser. **a**, AlphaFold-predicted structure of FENG8, with colors indicating pLDDT confidence scores (upper panel). **b**, N-terminal domain of FENG8 (pink), with a disordered region of 264 amino acids highlighted in blue. **c**, Structural comparison of the N-terminal domain in FENG8 (pink) and Helraiser (cyan). **d**, Structural comparison of the HUH domain in FENG8 (pink) and Helraiser (cyan). **e**, Structural comparison of the helicase domain in FENG8 (pink) and Helraiser (cyan).

The structure predicted by AlphaFold2 (ref. ^34^) revealed the presence of an ND and a helicase domain connected by two intermediate domains, INT1 and INT2, as observed in the experimentally determined structure of Helraiser, and similarly forming a large, tightly knit molecule (Fig. 1e and Extended Data Fig. 6a). The first 264 amino acids of the ND are predicted to be disordered except for two α-helices (Extended Data Fig. 6b). This is consistent with the cryo-electron microscopy structure of Helraiser, where the first 110 amino acids were found to be disordered^33^. Given the amino acid identity (33.92%) between FENG8 and Helraiser, the predicted and experimental protein structures both exhibited high overall similarity, suggesting a similar function (Extended Data Fig. 6c–e).

**Extended Data Fig. 7:**
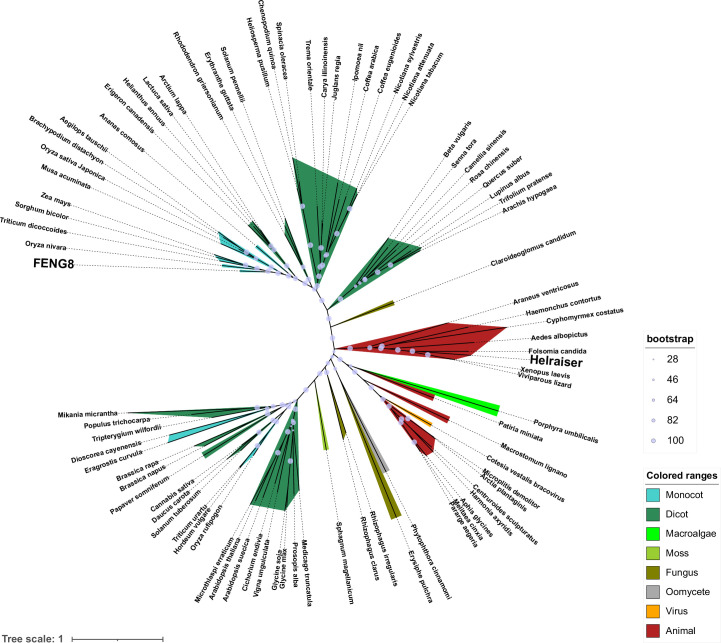
Phylogeny of FENG8 and other Helitron-like transposases. Maximum-likelihood phylogeny of the FENG8 transposase with Helitron-like transposases from 84 species. Detailed information for each protein can be found in the Supplementary Table 2.

To investigate the prevalence of the FENG8 transposase in other species, we extracted a total of 83 Helitron-like element coding sequences from the NCBI database plus Helraiser (Supplementary Table 2). The phylogenetic tree indicated that FENG8 is more closely related to Helitrons present in monocotyledonous plants, whereas Helraiser clustered with animal and insect sequences, suggesting a long-standing evolutionary divergence (Extended Data Fig. 7).

**Fig. 2:**
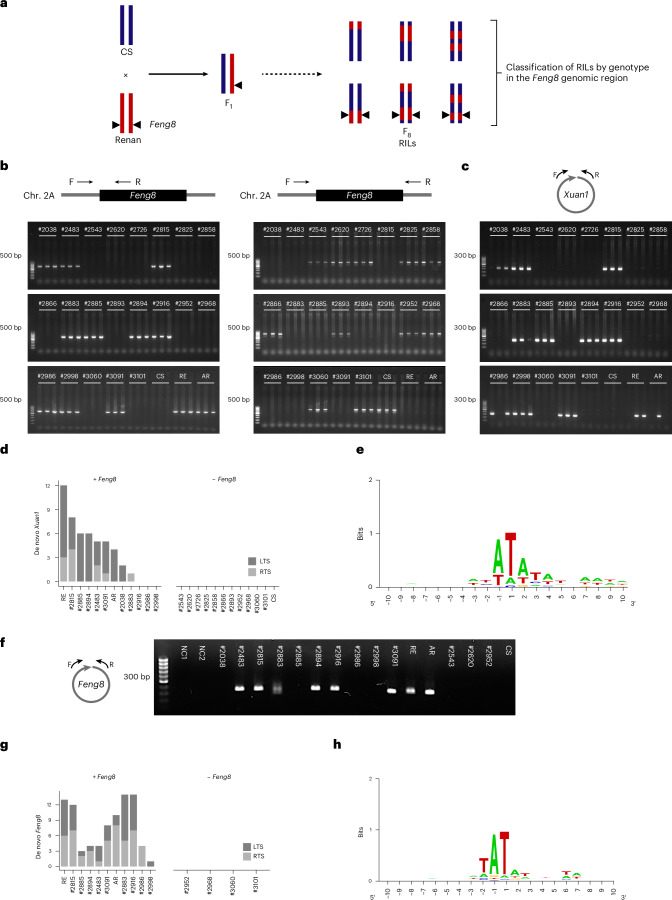
*Feng8* is necessary for *Xuan1* eccDNA biosynthesis and de novo insertion. **a**, The crossing scheme used to obtain CS × Renan RILs. The blue segments highlight chromosome segments originating from CS and the red ones those originating from Renan. The black triangles indicate the presence of *Feng8*. Each RIL was first analysed by SNP genotyping, and then the presence or absence of *Feng8* was confirmed by PCR (see **c**). **b**, *Feng8* genotyping by PCR for the presence (left) and absence (right) of the Helitron. The first lane shows the GeneRuler 100-bp DNA ladder. RE, Renan; AR, Arina. **c**, Inverse PCR detection of *Xuan1* eccDNA for each JZH-treated RIL (indicated by the numbers at the top) with three biological replicates. The first lane shows the GeneRuler 100-bp DNA ladder. **d**, Quantification of somatic *Xuan1* de novo insertion events in the genomes of RILs that have *Feng8* (+) and lines that do not (−). **e**, Sequence logo of 49 de novo *Xuan1* integration sites (the insertion site is between the −1 and 1 base positions). **f**, Inverse PCR detection of *Feng8* eccDNA for each JZH-treated RIL (indicated by the numbers at the top). The first lane shows the GeneRuler 100-bp DNA ladder. NC, non-DNA control. **g**, Quantification of somatic *Feng8* de novo insertion events in the genomes of RILs that have *Feng8* (+) and lines that do not (−). **h**, Sequence logo of 86 de novo *Feng8* integration sites (the insertion site is between the −1 and 1 base positions). Source data

**Extended Data Fig. 8:**
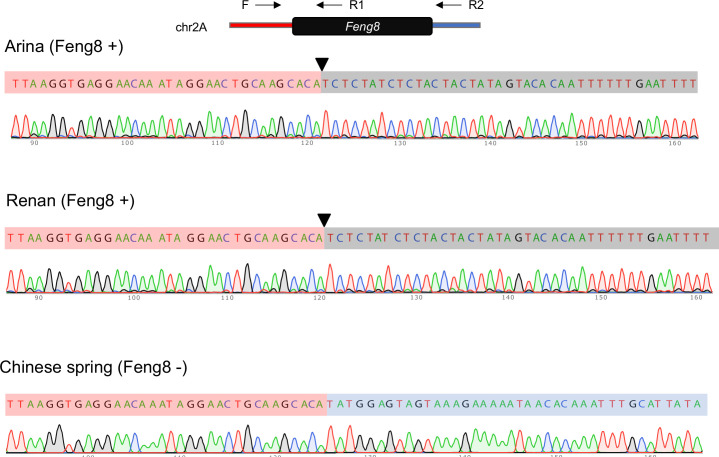
Sanger validation of the *Feng8* insertion site in three wheat cultivars. Sanger sequencing chromatograms spanning the *Feng8* insertion locus in three wheat cultivars. Arina and Renan show continuous sequence across the upstream flank (red background), *Feng8* insertion (gray background) confirming the presence of the insertion. In contrast, Chinese Spring lacks the *Feng8* sequence at this locus and shows direct junction of upstream (red background) and downstream flanking (blue background) regions. The insertion breakpoint is indicated as black triangle.

We next wanted to genetically test whether *Xuan1* mobilization requires *Feng8*. To achieve this, we took advantage of the contrasting responses of two wheat varieties to the JZH treatments: first, the variety CS, in which *Feng8* is absent^35^ and which did not spawn *Xuan1* eccDNA following JZH treatment (Fig. 1b); and second, the Renan cultivar, which does carry an intact *Feng8* copy in its genome^36^ and which spawned *Xuan1* eccDNAs upon JZH treatment (Fig. 1b and Extended Data Fig. 8). We cloned and sequenced *Feng8* from the Arina and Renan genomic DNA and found that they showed 100% identity in the nucleotide sequence. Similarly, our comparison of the *Xuan1* sequences of the Arina, CS and Renan varieties showed 100% identity. To genetically test the requirement of *Feng8* for *Xuan1* mobilization, we took advantage of wheat recombinant inbred lines (RILs) that were obtained from an initial cross of CS and Renan followed by eight generations of self-pollination (Fig. 2a). First, on the basis of single-nucleotide polymorphism (SNP) genotyping data, 21 RILs were selected: 10 lines with *Feng8* (the Renan allele in the region) and 11 lines without (the CS allele in the region). The presence or absence of *Feng8* was further confirmed by PCR for all RILs (Fig. 2b). To test the capacity of these RILs to produce *Xuan1* eccDNA, they were all subjected to JZH treatment. We found that only the RILs with *Feng8* produced *Xuan1* eccDNA, as confirmed by inverse PCR (Fig. 2c). We therefore demonstrate a genetic association between *Feng8* and the induction of *Xuan1* eccDNA biosynthesis. As eccDNA levels were very low in these lines, we consistently performed these assays with three biological replicates, and we never detected false positives.

**Extended Data Fig. 9:**
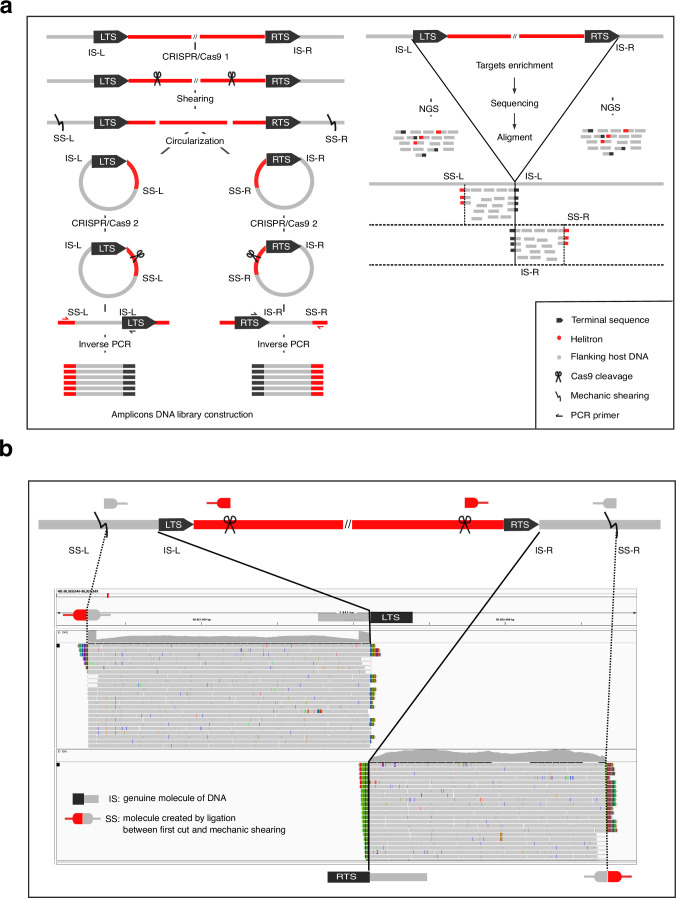
Identification of *de novo Helitron* insertions using PCIP-seq. **a**, Graphical representation of the PCIP-seq procedures. Genomic DNA is specifically cut with a CRISPR/Cas9 targeting the *Helitron* genomic sequence. The DNA is then mechanically sheared, generating random left and right shearing sites (SS-L and SS-R). The DNA is then end-repaired, circularized, and cut again by a second set of two CRISPR/Cas9 targeting *Helitron* sequences adjacent to *Helitron* LTS and RTS to specifically reopen circles encompassing left and right insertion sites (IS-L and IS-R) in two separated pools (left terminal site [LTS] and right terminal site [RTS]). Fragments encompassing the IS and SS are then amplified by long-range inverse PCR using divergent *Helitron* targeting primer pairs. The resulting PCR products were then subjected to next generation sequencing (NGS) and the obtained sequence reads were mapped on the Chinese Spring IWGSC RefSeq^5^, revealing *Helitron* IS and their corresponding SS that allow the precise and sensitive identification of somatic *Helitron* insertions. **b**, Integrative Genome Viewer (IGV) screen capture of a sense *de novo Feng8* insertion captured in sample (#2883) in both LTS and RTS pools using PCIP-seq^38^.

Next, we wanted to test whether *Xuan1* and *Feng8* can complete their life cycle by integrating de novo into the wheat genome. To capture somatic de novo insertions of *Xuan1* and *Feng8* in the RILs, we harnessed the high sensitivity of the well-established pooled CRISPR inverse PCR sequencing method (PCIP-seq) (Methods and Extended Data Fig. 9a). This method has been demonstrated to efficiently detect rare somatic genomic insertions of exogenous and endogenous viruses (including TEs) in humans, cattle and sheep^37,38^. We blindly applied this method to the 21 RILs to detect novel somatic *Xuan1* insertions. We captured a total of 49 somatic *Xuan1* de novo insertions in 8 out of 10 RILs that carried *Feng8* and none in the 11 RILs without *Feng8* (Fig. 2d and Supplementary Table 3). We extracted 20 bp of flanking wheat host DNA sequence at these 49 de novo insertion sites and found a marked preference for insertions at AT dinucleotides (Fig. 2e and Supplementary Table 3), an expected feature for bona fide Helitron insertions. To test whether *Feng8* itself was also circularized and mobilized, we applied inverse PCR to detect *Feng8* eccDNA and PCIP-seq to detect somatic *Feng8* de novo insertions in the RILs and the parental lines. We found that *Feng8* also produced eccDNA (Fig. 2f) and was mobile, as we detected 86 de novo insertions in the *Feng8*-carrying lines (Fig. 2g) and none in the lines that do not have *Feng8*. Again, we found a marked insertion preference at AT dinucleotides, demonstrating the specificity of the method (Fig. 2h). For all *Xuan1* and almost all *Feng8* insertions, we could detect either the LTS or RTS junction but not both for the same insertion event. That could be expected as we sampled the plants shortly after stress, thus following few cell divisions. Still, for one *Feng8* insertion, we managed to capture both junctions (Extended Data Fig. 9b and Supplementary Table 3). From that we conclude that *Xuan1* and *Feng8* insertions are rare and predominantly existed in only a single cell, thus providing only a single DNA fragment available for capture. Notably, we found that both *Xuan1* and *Feng8* integrated in the vicinity of or within genes in 34.6% of the cases (Supplementary Table 3). To assess the heritability of *Xuan1* or *Feng8* insertions to the next generation, we tested 99 plants descending from 6 JZH-treated mother plants for novel insertions. None of the progeny plants had inherited novel *Xuan1* or *Feng8* insertions, suggesting that the transmission of newly induced insertions to the next generation did not occur, or occurs only rarely under the conditions tested.

**Fig. 3:**
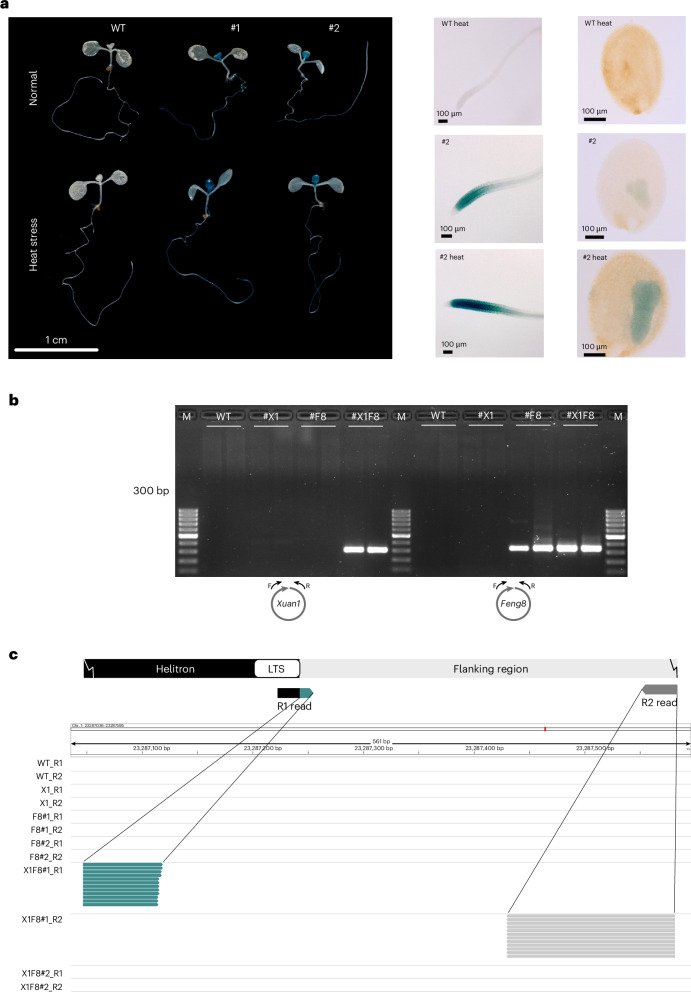
Heat-responsive activation of *Feng8* and functional reconstitution of *Xuan*–*Feng* reactivation in *Arabidopsis*. **a**, 966 bp of the *Feng8* promoter fused to GUS drives basal expression under control conditions (21 °C) and shows markedly enhanced and broader staining following heat treatment (37 °C). Representative whole-seedling images (left) and magnified views of roots and embryos (right) are shown. Two independent lines with three biological replicates were tested. **b**, Detection of *Xuan1* and *Feng8* eccDNA by inverse PCR in wild type (WT) and transgenic *Arabidopsis* lines expressing *Xuan1* alone (#X1), *Feng8* alone (#F8) or both (#X1F8) after heat treatment. Two independent lines with three biological replicates were tested. The M lanes show the GeneRuler 100-bp DNA ladder. **c**, TEd-seq detection of a representative de novo insertion event in *Arabidopsis*. Reads spanning the *Feng8* LTS and flanking genomic DNA are shown. High-confidence paired-end reads (R1 and R2) support insertion junctions in X1F8#1 lines, whereas no insertions are detected in WT or other lines.

To further characterize the heat responsiveness of *Feng8*, 966 bp of its promoter region was fused to the β-glucuronidase (GUS) reporter gene and transformed into *Arabidopsis thaliana*. Under control conditions (21 °C), GUS activity was detected primarily in vascular tissues, the shoot apical region and embryonic tissues. Upon heat treatment at 37 °C, GUS staining intensity increased markedly and became more broadly distributed across tissues (Fig. 3a). These results indicate that the *Feng8* promoter is temperature-responsive and sufficient to drive transcriptional activation under heat stress.

To directly test whether *Feng8* is sufficient to drive *Xuan1* mobilization, we generated independent transgenic *Arabidopsis* lines containing (1) *Xuan1* alone (#X1), (2) *Feng8* alone (#F8) and (3) *Xuan1* together with *Feng8* (#X1F8). On the basis of the promoter assay results, all lines were subjected to heat treatment at 37 °C to induce *Feng8* expression. We first examined eccDNA formation by inverse PCR. #X1 lines did not produce detectable *Xuan1*-derived eccDNA. In contrast, #F8 lines showed robust *Feng8*-derived eccDNA formation, and #X1F8 lines produced both *Xuan1* and *Feng8* eccDNA (Fig. 3b). To determine whether eccDNA formation corresponds to bona fide somatic transposition events and to employ a complementary sequencing approach to PCIP, we used the transposable element display sequencing (TEd-seq) protocol to detect somatic insertions^39^. We identified one *Xuan1* insertion and three *Feng8* insertions supported by high-confidence paired-end reads, all occurring at AT dinucleotides. In addition, we manually inspected the full R1 reads and confirmed the presence of the expected Helitron terminal sequence at the insertion junctions (Fig. 3c and Supplementary Table 4). A de novo *Xuan1* insertion was detected only in the #X1F8 line, whereas *Feng8* insertions were detected in both the #F8 and #X1F8 lines. These results indicate that *Xuan1* and *Feng8* can complete their life cycle in a heterologous system, albeit at a very low frequency.

In summary, our study demonstrates that an epigenetically silenced and heat-stress-responsive Helitron can be activated in wheat. This observation is evidenced by transcriptional activity, eccDNA formation and the documentation of novel somatic insertions. Genetic segregation in wheat RILs established a strict association between the presence of *Feng8* and the mobilization of *Xuan1*. This was further supported by heterologous reconstitution in *Arabidopsis*. This demonstrates that *Feng8* is required and sufficient to drive *Xuan1* mobilization, thereby confirming that the *Xuan*–*Feng* Helitron family relies on *Feng8* as its sole fully intact autonomous element. Together, our findings provide insights into TE biology in wheat and Helitron mobility in general.

## Methods

### Plant materials and growth conditions

All cultivars of wheat seeds (Arina, CH Nara, Rosatch, CS and Norin61) were obtained from Agroscope GenBank. RILs were obtained from INRAE GenBank. Prior to planting, the wheat seeds were soaked overnight in sterilized water and subsequently sterilized using a 10-min heat shock at 50 °C^40^. Following surface sterilization, three seeds were planted on 10 ml of agar media (0.55%) in a PHCbi MLR-352 growth chamber. The growth chamber was set to long-day conditions, with 16 h of light at 20 °C during the day and 18 °C at night, and a light level of 4 was maintained throughout the experiment. For *Arabidopsis*, seeds were surface sterilized with 70% ethanol and washed three times with sterile water. Sterilized seeds were sown on 1/2 Murashige and Skoog plates, stratified for two days at 4 °C and germinated under long-day conditions (16 h light / 8 h darkness) at 21 °C.

### Chemical treatment and stress

The treatment group seeds were cultured on agar media containing two drugs (as suggested by epibreed AG)—namely, 50 µM Juglone (Sigma, H47003) and 40 µM zebularine (Sigma, Z4775) dissolved in DMSO. An equal volume of DMSO (Fisher Scientific, BP231-100) was administered to the control group seeds. Heat stress was induced by exposing six-day-old seedlings to 40 °C for 24 h. For *Arabidopsis*, heat stress was induced by exposing six-day-old seedlings to 37 °C for 24 h.

### DNA extraction and eccDNA sequencing

For wheat samples, the uppermost 3-cm section of the stem (coleoptile) was harvested from three individual seedlings, and the coleoptile sheath was carefully removed. The three harvested shoot sections were combined into one tube immediately at the end of the stress period and snap-frozen in liquid nitrogen, followed by storage at −80 °C until DNA extraction. For *Arabidopsis* samples, around 25 whole seedlings were collected for one tube in −80 °C after treatment until DNA extraction. The total DNA was then extracted from the frozen samples using the modified CTAB method^41^.

The eccDNA-seq method was employed for all samples, as previously described^42^. Briefly, 2 µg of total DNA was digested with 10 U of PlasmidSafe (LubioScience, E3101K) for 16 h at 37 °C to eliminate linear DNA, followed by enzyme denaturation at 70 °C for 30 min. Next, 100 ng of the digested DNA was precipitated with ethanol supplemented with 1 µl of GlycoBlue (Fisher Scientific, 10391565). Circular DNA was then amplified through RCA using the Illustra TempliPhi kit (GE Healthcare, 25-6400-10) according to the manufacturer’s instructions, with the reaction left for 16 h at 30 °C. The RCA reaction was inactivated by heating at 65 °C for 10 min, followed by cooling at 4 °C. The amplified DNA was once again precipitated with ethanol and debranched using 10 U of T7 Endonuclease I (New England Biolabs, M0302S) by incubating at 37 °C for 30 min. After ethanol precipitation, 400 ng of debranched DNA was used for library preparation with the Nanopore Rapid Barcoding Sequencing Kit (SQK-RBK004). The eccDNA-seq was carried out using PromethION 2 Solo with flow cell FLO-PRO114M, and basecalling was performed using Guppy v.7.1.4 (ref. ^43^).

### Wheat whole-genome sequencing

The high-molecular-weight genomic DNA from the leaf samples was fragmented using a gTube to select fragments of approximately 20 kb. The library preparation was carried out using a ligation sequencing kit (SQK-LSK109), and sequencing was performed on the FLO-PRO002 flow cell of PromethION by Biomarker (BMKGENE) Europe. The clean data obtained from Nanopore sequencing were aligned to the wheat reference genome (Triticum_aestivum_Arina*LrFor*_v3.0, IPK) using minimap2 (ref. ^44^) v.2.26 with the default parameters. Subsequently, we employed nanomonsv^45^ v.0.7 to detect structural variations.

### Whole-genome DNA methylation analysis

High-molecular-weight genomic DNA was extracted from coleoptile tissues and used for library construction. Sequencing libraries were prepared using the Oxford Nanopore Technologies Ligation Sequencing Kit V14 (SQK-LSK114) following the manufacturer’s instructions. Libraries were sequenced on an ONT PromethION 2 platform using FLO-PRO114M flow cells, generating approximately 11.15× (wild type) and 12.92× (JZH-treated) genome coverage. Basecalling and modified base detection were performed using Dorado v.0.7.11.2 (ref. ^46^) with a high-accuracy model enabling simultaneous detection of 5-methylcytosine (5mC) across all sequence contexts. To ensure accurate mapping of *Feng8*-associated reads, the *Feng8* sequence was manually inserted into the Arina*LrFor* reference assembly at chromosome 2A: 319212266–319213602. Raw reads were aligned to the modified reference using minimap2 v.2.29 (parameters: -ax lr:hq; -2; -y; -secondary=no). Methylation calls were aggregated using modkit v.0.6.1 with the modkit pileup function, specifying the detection of 5mC and 5hmC. Methylation levels were calculated separately for CG, CHG and CHH contexts using motif-specific parameters (-motif CG, 0; -motif CHG, 0; -motif CHH, 0) and combined strand information. Differential methylation analysis at single-base resolution was performed using the modkit dmr pair function with parameters -base C -fine-grained.

### eccDNA loci identification

Basecalled, demultiplexed and trimmed eccDNA-seq reads from the same sample were concatenated. To identify eccDNA formation regions in JZH-treated Arina seedlings, the reads were aligned to the wheat reference genome (Triticum_aestivum_Arina*LrFor*_v3.0, IPK) using ecc_finder v.1.0.0 in map-ont mode with the parameter MAX_SIZE in the Genrich.h dependency script altered to 1,000,000 to enable the processing of long-read alignments.

### Inverse PCR to test for eccDNA circle junctions

A total of 100 ng of genomic DNA and 50 ng of RCA-amplified DNA were used as separate templates to generate inverse PCR. Specific primers were designed to span the junction of the *Xuan1* and *Feng8* circles. The PCR reaction was performed in a 20-µl volume according to the manufacturer’s protocol of GoTaq G2 DNA Polymerase (Promega, M7841). The PCR amplification conditions consisted of an initial denaturation step at 95 °C for 2 min, followed by 30 cycles of denaturation at 94 °C for 30 s, annealing at 58 °C for 30 s, extension at 72 °C for 2 min and a final extension step at 72 °C for 10 min. The purification of PCR products was carried out using the Wizard PCR clean-up system (Promega, A9281), following the Sanger sequencing service provided by Microsynth AG.

### Helitron annotation and *Xuan*–*Feng* family identity

For Helitron annotation, we filtered all candidate sequences identified by HELIANO^47^ v.1.2.0 using the parameters -pt 1 -is1 1 -is2 1 -p 1e-5. To identify members of the *Xuan*–*Feng* family, we used the LTS–RTS of *Xuan1* as the reference LTS–RTS pair file for the HELIANO ts parameter, applying -dis_denovo -nearest -dn 8000 for dis-denovo prediction. The resulting sequences were then manually curated by excluding those containing ‘N’ bases and selecting 30 bp of the LTS and the RTS to obtain high-confidence members.

### Transcript analysis

Total RNA extractions were carried out using the NucleoSpin RNA kit (Macherey-Nagel, 740955.50) for three biological replicate samples under each condition. The extracted RNA samples were subjected to Illumina 150-bp paired-end sequencing using a stranded poly-A directional library at Novogene. Raw mRNA-seq reads were aligned to the complete gene sequences of *Feng1-8* and *Xuan1* using STAR^48^ v.2.7.10a with the parameter outFilterMismatchNmax set to 0 to disallow mismatches. Alignment BAM files were filtered with samtools^49^ v.1.18 to keep only primary mappings and were normalized and converted to bigwig format with the scale function of BAMscale^50^ v.1.0 using the parameters -operation rna -scale custom and -factor with custom normalization factors for each library. Normalization factors were calculated by assembling a de novo reference transcriptome with Trinity^51^ v.2.15.1 using all reads and the default parameters, quantifying transcripts with RSEM^52^ v.1.3.3 and bowtie2 (ref. ^53^) as aligners and applying the function calcNormFactors from the R library edgeR^54^ v.4.0.3 on the posterior mean counts in R v.4.3.2 (ref. ^55^).

### Cloning and sequencing of PCR fragments

*Feng8* cloning from Arina and Renan genomic DNA was produced by PCR using the TOPO XL-2 complete PCR cloning kit (Invitrogen, K805010 and K805020). The purified PCR products were ligated with the pCR-XL-2-TOPO vector at a molar ratio of 1:1 (transcript:vector). The ligation reaction was carried out at 25 °C for 30 min. Subsequently, 2 µl of the ligation product was transformed into One Shot OmniMAX 2 T1^R^ chemically competent *Escherichia coli* using a heat-shock method. The transformed cells were plated on selective agar plates, and individual colonies were selected for further analysis. The presence of the desired inserts in the colonies was confirmed by PCR, and the plasmids were subsequently purified using the PureLink quick plasmid miniprep kit (Invitrogen, K210010). To ensure the integrity of the cloned inserts, the entire plasmid sequence was determined using Microsynth AG.

### Transposase analyses and structure prediction

The 3D structure prediction of the protein was obtained using AlphaFold^34^ v.2.3.1. Five parallel model predictions were conducted for each sequence, and the structures with the highest predicted local distance difference test scores were selected for analysis and visualization using PyMOL v.2.1 (ref. ^56^).

### Phylogenetic tree construction

To generate the maximum-likelihood phylogenetic tree for transposase, the amino acid sequence of FENG8 was employed as the query for a blastp search against the NCBI database. Top-scoring hits from 83 different species were chosen to construct the phylogenetic tree. Multiple sequence alignment was carried out using MUSCLE^57^ v.5.1, and the alignment was subsequently converted to phylip format using trimAl^58^ v.1.4. The phylogenetic tree was constructed using IQ-TREE^59^ v.2.12 with a bootstrap value of 1,000 replicates.

### Selection of 21 CS × Renan RILs based on SNP genotyping data

We analysed SNP genotyping data (Axiom array TaBW420k) available for 282 individuals originating from a cross between CS and Renan^60^, to select 21 individuals that carry either CS or Renan alleles at both *Feng8* (chromosome 2A) and *Xuan1* (chromosome 6D) loci. The positions of the markers were identified by mapping SNP context sequences on the CS IWGSC RefSeq v.2.1 using the pipeline nf_remapMarkers (https://forgemia.inra.fr/umr-gdec/nf-remapmarkers). Although *Feng8* is absent from CS, its insertion site is at position 323357927 on chromosome 2A. The position of *Xuan1* is 169657404 on chromosome 6D. We retrieved SNP alleles in a window of 100 Mb centred on each of the two loci on chromosomes 2A and 6D. This represented 496 and 163 SNPs on these two chromosomes, respectively. We kept only 132 individuals, all alleles within 100 Mb, encompassing each locus derived from the same parent. We classified the 132 individuals into four haplotypes: A/A, A/B, B/B and B/A (allele A is from CS, and allele B is from Renan; *Feng8*/*Xuan1*). B/B and B/A individuals were expected to carry a functional *Feng8* originating from the Renan parent, while *Feng8* was predicted to be absent for A/B and A/A individuals. We eventually randomly selected 21 individuals for their ability to produce eccDNAs so that 5, 5, 5 and 6 were of the following haplotypes: B/B (#2038, #2483, #2883, #2986 and #2998), B/A (#2815, #2885, #2894, #2916 and #3091), A/B (#3101, #2620, #2866, #2893 and #3060) and A/A (#2968, #2726, #2825, #2858, #2952 and #2543), respectively.

### Modified PCIP-seq to capture de novo Helitron insertions

The method has been described in detail in Tang et al.^38^. The single-guide RNAs (sgRNAs) and primers were designed on the basis of the *Xuan1* and *Feng8* sequences. The experimental protocols and data analysis procedures were identical for *Xuan1* and *Feng8*, except for the initial cleavage: one sgRNA (1,357 bp from the LTS) targeted *Xuan1*, while two sgRNAs were used to cut two sites in *Feng8* (1,222 bp from the LTS and 1,068 bp from the RTS). The description below uses *Xuan1* as an example.

#### Molecular biology

Using 500 ng of genomic DNA as starting material, fragments containing *Xuan1* sequences were cleaved using sgRNAs (Integrated DNA Technologies) targeting sequences at 1,357 bp from the LTS and *S. pyogenes* Cas9 (New England Biolabs, M0386S). The digested DNA was further mechanically sheared to ∼3 kb using a Megaruptor-1 (Diagenode), end-repaired using a NEBNext EndRepair Module (New England Biolabs, E6050L) and purified with Agencourt AMPure XP beads (Beckman Coulter, A63881). The resulting DNA fragments were circularized using T4 DNA Ligase (New England Biolabs, M0202L), residual linear fragments were eliminated with Plasmid-Safe-ATP-Dependent DNase (Epicentre, E3110K), the remaining fragments were purified and reaction products were split in two aliquots. Circular molecules encompassing *Xuan1* sequences were then reopened with *S. pyogenes* Cas9 using distinct sgRNAs for the two and purified. Encompassing linear fragments were inverse-PCR-amplified using aliquot-specific primer pairs with LongAmp Taq DNA Polymerase (New England Biolabs, M0533S) and purified. The purified amplicons were mechanically sheared to ∼350 bp using a Bioruptor-pico (Diagenode), sequencing libraries were generated using the NEBNext Ultra II DNA Library Prep Kit for Illumina (New England Biolabs, E7645L) and indexed libraries were pooled and sequenced on a Novaseq 6000 sequencer (Illumina) targeting ∼8 million 150-bp paired-end reads per library.

#### Data processing

The ensuing sequence reads were demultiplexed, the reads were quality assessed using fastQC^61^, adapter sequences were trimmed using Cutadapt^62^ and trimmed reads were mapped to the CS IWGSC RefSeq genome^5^ using BWA-MEM^63^ and converted to BAM format using SAMtools^64^. Using a custom-made Python script, we first identified clipped reads using CIGAR information. We selected clipped reads with mapping quality ≥40 and a minimum of 10 clipped bases. We then mapped the clipped reads to the segments of the corresponding *Xuan1* genome. We specified an alignment score ≥0.6 to declare a hit and labelled the read as either an insertion site (IS) or a shearing site (SS) read. When possible, we extended the alignment with *Xuan1* into non-clipped bases to refine the positions of the SS and IS. We then merged SS and IS sites with the same ‘breakpoint’, thereby identifying candidate SS and IS supported by multiple concordant reads. We then paired the IS with their cognate SS. The pairing was based on orientation (for example, a 5′ SS should be located upstream of the 5′ IS for a *Xuan1* element in sense orientation, and downstream of the 5′ IS for a *Xuan1* in antisense orientation) and distance (the maximum distance between IS and SS was set at 5 kb).

### Vector construction and *Arabidopsis* transformation

To generate the GUS reporter lines, the first 966-bp fragment of *Feng8* before the translation start site was amplified and used as the putative promoter sequence. The fragment was cloned upstream of the GUS reporter gene in a modified pGWB553 vector. The *Xuan1* element was amplified and inserted into the pGAPBRHyg vector at the SpeI restriction site without an additional promoter sequence. The full-length *Feng8* sequence was introduced into the pBGW0 binary vector using Gateway recombination (Invitrogen) according to the manufacturer’s instructions. Subsequently, the *Xuan1* element was inserted into the pBGW0-Feng8 construct at the SbfI site using the In-Fusion HD Cloning system (Takara) to generate the pBGW0-Xuan1_Feng8 construct. No additional promoter sequence was included in this construct. All constructs were verified by Sanger sequencing prior to transformation. For plant transformation, constructs were introduced into *Agrobacterium tumefaciens* strain GV3101 by electroporation. *Arabidopsis* (Col-0 ecotype) plants were transformed using the floral dip method. Transgenic plants were selected on Murashige and Skoog medium supplemented with the appropriate antibiotics.

### GUS staining assay

Six-day-old seedlings were divided into control and heat-treatment groups. Control seedlings were maintained at 21 °C, whereas heat-treated seedlings were incubated at 37 °C for 12 h under identical continuous light conditions. Following treatment, seedlings were subjected to histochemical GUS staining. The samples were incubated in GUS staining buffer containing 50 mM sodium phosphate buffer (pH 7.0), 10 mM EDTA, 0.1% (v/v) Triton X-100, 1 mM potassium ferrocyanide (K_4_Fe(CN)_6_), 1 mM potassium ferricyanide (K_3_Fe(CN)_6_) and 1 mg ml^−1^ 5-bromo-4-chloro-3-indolyl β-D-glucuronic acid (X-Gluc). The seedlings were incubated at 20 °C for 4 h. After staining, tissues were cleared in 70% (v/v) ethanol until chlorophyll was fully removed. For seed analysis, seeds were mounted in Hoyer’s solution on microscope slides and incubated at room temperature for four days prior to imaging.

### TEd-seq library construction and analysis

TEd-seq libraries were constructed with modifications from Vendrell-Mir et al.^39^. Briefly, 1 µg genomic DNA was sheared to 250–500 bp using a Bioruptor Plus (three cycles of 15 s on, 30 s off). Fragmented DNA was end-repaired and A-tailed using the NEBNext Ultra II End Prep Enzyme Mix, followed by ligation of TEd-seq forked adapters. Adapter-ligated DNA was size-selected with AMPure XP beads and subjected to selective amplification of TE-containing fragments using NEBNext Ultra II Q5 Master Mix and outer primers (TEDseq_outer_for and TE_outer_rev). PCR products were purified and size-selected (0.9× beads), followed by a second round of nested PCR incorporating custom Illumina P5 and indexed P7 adapters. Final libraries were purified (0.9× beads), eluted in 10 mM Tris-HCl and sequenced (paired-end, 300 cycles) on a NextSeq 1000 platform.

Paired-end reads were analysed using a custom pipeline to identify genomic Helitron insertion sites. In this design, Read 1 (R1) corresponds to the TE-adjacent end, whereas Read 2 (R2) represents the paired genomic flank. Reads were first filtered using TE-specific primers (24 bp) with full-length exact matching in cutadapt (v.5.2). Primer-positive reads were subsequently filtered using TE extremity sequences (10 bp for *Xuan1* and 14 bp for *Feng8*), also requiring full-length exact matches. Extremity sequences were trimmed, and only read pairs retaining ≥15 bp of genomic sequence were retained. Trimmed R1 reads (R1_ext) were mapped to the TAIR10 reference genome using BWA-MEM (v.0.7.18). Only fully aligned reads without soft or hard clipping were considered. The genomic alignment start position of each R1_ext read was defined as the insertion coordinate. Trimmed R2 reads were independently mapped, and only fully aligned reads were retained. R1-derived insertion candidates were required to have R2 support within ±400 bp to ensure paired-end consistency. Insertions overlapping within 1 kb across samples were excluded from the analyses.

All primers used for this study are listed in Supplementary Table 5.

### Reporting summary

Further information on research design is available in the Nature Portfolio Reporting Summary linked to this article.

## Supplementary information

Reporting Summary

Supplementary Table 1 The *Xuan*–*Feng* Helitron family members in the Arina*LrFor* genome.

Supplementary Table 2 The 83 Helitron-like proteins identified by homology search.

Supplementary Table 3 Detailed information related to the 49 de novo *Xuan1* and 86 de novo *Feng8* insertions captured by PCIP-seq.

Supplementary Table 4 Detailed information related to the *Xuan1* and *Feng8* insertions captured by TEd-seq in *Arabidopsis*.

Supplementary Table 5 Primers used in this study.

## Source data

Source Data Fig. 2 Unprocessed gels.

Source Data Extended Data Fig. 4 Statistical source data.

## Data Availability

The *Xuan1* and *Feng8* sequences have been submitted to NCBI GenBank with accession numbers PP376094 and PP376095. The sequencing data from this study have been submitted to the European Nucleotide Archive (www.ebi.ac.uk/ena/, accession number ERP157467) under project number PRJEB72688. The raw whole-genome sequencing reads are under fastq accessions ERR12723706–ERR12723708, and the raw eccDNA-Seq reads for Arina are under the accessions ERR12724408–ERR12724413. The raw RNA-seq reads are under the accessions ERR12724677–ERR12724682. Positions of TaBW420K SNP markers on CS IWGSC RefSeq v.2.1 are available at 10.57745/YXNWZK. The raw fastq files for the PCIP-seq experiments applied to the 24 RILs have been submitted to the European Nucleotide Archive under project number PRJEB75199. The raw fastq files for the TEd-seq experiments applied to *Arabidopsis* lines have been submitted to the European Nucleotide Archive under project number PRJEB108718. Source data are provided with this paper.

## Code availability

The pipeline for identifying genomic Helitron insertion sites is available without restriction via GitHub at https://github.com/Lijingtangbo/TE-rate_manuscript.
