# Supplementary material for: An active Helitron transposon family in wheat
Source: Nat Plants. 2026 Jun 5;12(6):1209–19. doi: 10.1038/s41477-026-02319-3 (PMC13286980; doi:10.1038/s41477-026-02319-3)
Supplement: Supplementary file 1 — Reporting Summary [file 41477_2026_2319_MOESM1_ESM.pdf]

nature portfolio

Corresponding author(s): Etienne Bucher

Last updated by author(s): Apr 30, 2026

Reporting Summary

Nature Portfolio wishes to improve the reproducibility of the work that we publish. This form provides structure for consistency and transparency in reporting. For further information on Nature Portfolio policies, see our Editorial Policies and the Editorial Policy Checklist.

Statistics

For all statistical analyses, confirm that the following items are present in the figure legend, table legend, main text, or Methods section.

n/a	Confirmed	
☐	☒	The exact sample size (*n*) for each experimental group/condition, given as a discrete number and unit of measurement
☐	☒	A statement on whether measurements were taken from distinct samples or whether the same sample was measured repeatedly
☐	☒	The statistical test(s) used AND whether they are one- or two-sided<br>*Only common tests should be described solely by name; describe more complex techniques in the Methods section.*
☒	☐	A description of all covariates tested
☒	☐	A description of any assumptions or corrections, such as tests of normality and adjustment for multiple comparisons
☒	☐	A full description of the statistical parameters including central tendency (e.g. means) or other basic estimates (e.g. regression coefficient) AND variation (e.g. standard deviation) or associated estimates of uncertainty (e.g. confidence intervals)
☐	☒	For null hypothesis testing, the test statistic (e.g. *F*, *t*, *r*) with confidence intervals, effect sizes, degrees of freedom and *P* value noted<br>*Give P values as exact values whenever suitable.*
☒	☐	For Bayesian analysis, information on the choice of priors and Markov chain Monte Carlo settings
☒	☐	For hierarchical and complex designs, identification of the appropriate level for tests and full reporting of outcomes
☒	☐	Estimates of effect sizes (e.g. Cohen's *d*, Pearson's *r*), indicating how they were calculated

Our web collection on statistics for biologists contains articles on many of the points above.

Software and code

Policy information about availability of computer code

Data collection	We used this open source software: Guppy v7.1.4 for basecalling
Data analysis	We used these software packages: minimap2 v2.26, nanomonsv v0.7, HELIANO, STAR v.2.7.10a, BAMscale, Trinity v.2.15.1, RSEM v.1.3.3, bowtie2, edgeR 7 v.4.0.3, R v.4.3.2, AlphaFold v2.3.1, PyMOL v2.1, MUSCLE v5.1, trimAl v1.4, IQ-TREE v2.12, BWA-MEM, SAMtools, Python code we wrote can be found here: https://github.com/Lijingtangbo

For manuscripts utilizing custom algorithms or software that are central to the research but not yet described in published literature, software must be made available to editors and reviewers. We strongly encourage code deposition in a community repository (e.g. GitHub). See the Nature Portfolio guidelines for submitting code & software for further information.

Data

Policy information about availability of data

All manuscripts must include a data availability statement. This statement should provide the following information, where applicable:

- Accession codes, unique identifiers, or web links for publicly available datasets
- A description of any restrictions on data availability
- For clinical datasets or third party data, please ensure that the statement adheres to our policy

The Xuan1 and Feng8 sequences have been submitted to NCBI GenBank with accession numbers PP376094 and PP376095. The sequencing data from this study have been submitted to the European Nucleotide Archive (ENA, www.ebi.ac.uk/ena/, accessed on ERP157467) under the project PRJEB72688. The whole genome

sequencing raw reads are under fastq accessions ERR12723706-ERR12723708, raw reads of eccDNA-Seq for Arina are under the accessions ERR12724408-ERR12724413. The raw reads of the RNA sequencing are under the accessions ERR12724677-ERR12724682. Positions of TaBW420K SNP markers on CS IWGSC RefSeq v2.1 are available under DOI: https://doi.org/10.57745/YXNWZK. The raw fastq files for the PCIP-seq experiments applied to the 24 RILs have been submitted to the European Nucleotide Archive (ENA, www.ebi.ac.uk/ena) under the projects PRJEB75199 and PRJEB108718

Research involving human participants, their data, or biological material

Policy information about studies with human participants or human data. See also policy information about sex, gender (identity/presentation), and sexual orientation and race, ethnicity and racism.

Reporting on sex and gender	N/A
Reporting on race, ethnicity, or other socially relevant groupings	N/A
Population characteristics	N/A
Recruitment	N/A
Ethics oversight	N/A

Note that full information on the approval of the study protocol must also be provided in the manuscript.

Field-specific reporting

Please select the one below that is the best fit for your research. If you are not sure, read the appropriate sections before making your selection.

☒ Life sciences ☐ Behavioural & social sciences ☐ Ecological, evolutionary & environmental sciences

For a reference copy of the document with all sections, see nature.com/documents/nr-reporting-summary-flat.pdf

Life sciences study design

All studies must disclose on these points even when the disclosure is negative.

Sample size	For the experiments, at least 3 biological replicates were used (including transcriptomics).
Data exclusions	No data was excluded from the analyses
Replication	All experiments were carried out at least twice and with at least 3 biological replicates
Randomization	does not apply
Blinding	The eccDNA experiments on the RILs and the Xuan-Feng insertion analyses by PCIP were carried out by blinding. Meaning that the person performing the experiments did not know the genotype of the tested RILs. The IDs of the RILS were only revealed after the results were obtained.

Reporting for specific materials, systems and methods

We require information from authors about some types of materials, experimental systems and methods used in many studies. Here, indicate whether each material, system or method listed is relevant to your study. If you are not sure if a list item applies to your research, read the appropriate section before selecting a response.

Materials & experimental systems

n/a	Involved in the study	
☒	☐	Antibodies
☒	☐	Eukaryotic cell lines
☒	☐	Palaeontology and archaeology
☒	☐	Animals and other organisms
☒	☐	Clinical data
☒	☐	Dual use research of concern
☐	☒	Plants

Methods

n/a	Involved in the study	
☒	☐	ChIP-seq
☒	☐	Flow cytometry
☒	☐	MRI-based neuroimaging

Plants

Seed stocks	All wheat varieties were obtained from the Agroscope National Gene Bank
Novel plant genotypes	Arabidopsis plants were transformed using Agrobacterium and binary vectors. From each constructs two independent transformant lines were tested in our experiments.
Authentication	Plants of the Agroscope National Gene Bank are genotyped. All RILs used were genotyped.
