# Supplementary figures and images for: An active Helitron transposon family in wheat
Source: Nat Plants. 2026 Jun 5;12(6):1209–19. doi: 10.1038/s41477-026-02319-3 (PMC13286980; doi:10.1038/s41477-026-02319-3)

## All unprocessed gels

Fig.1b

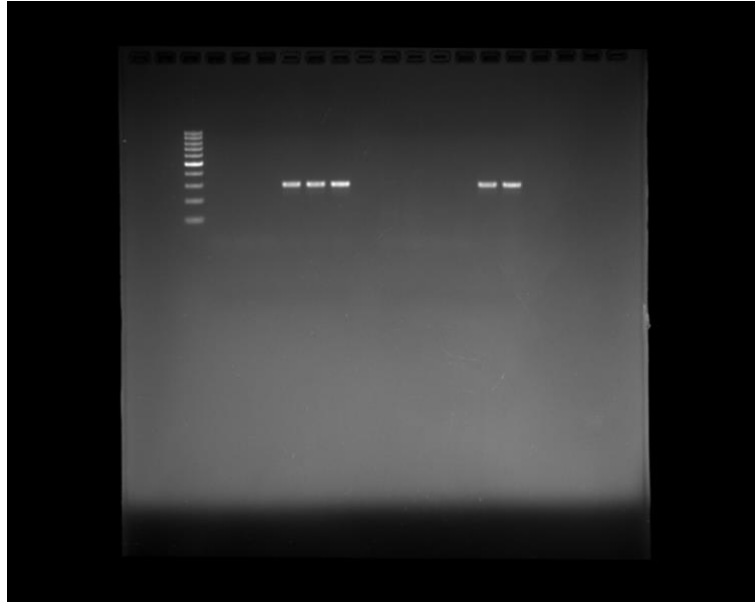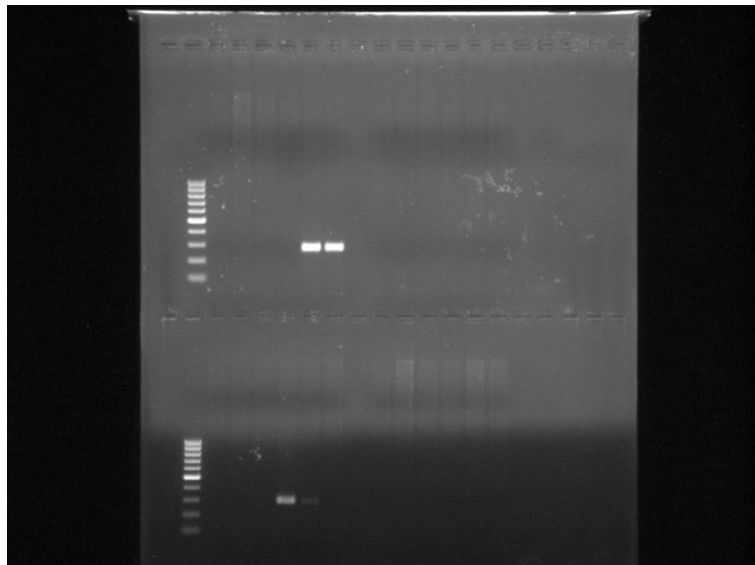

Fig.2b

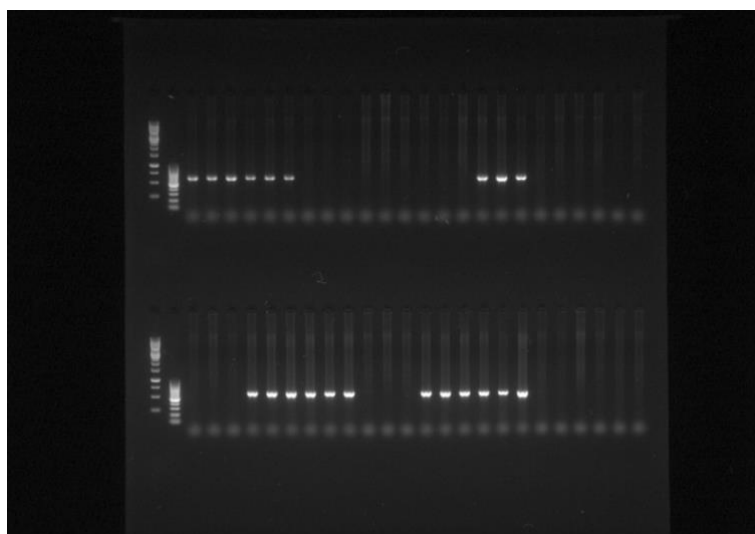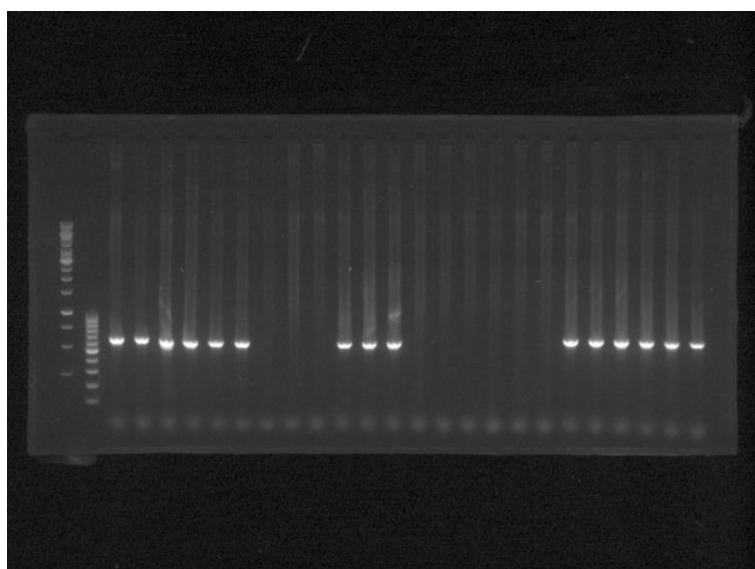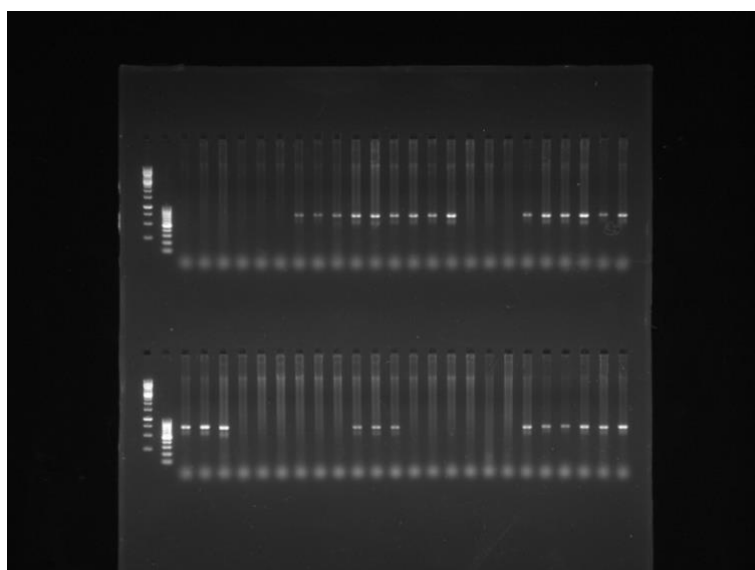

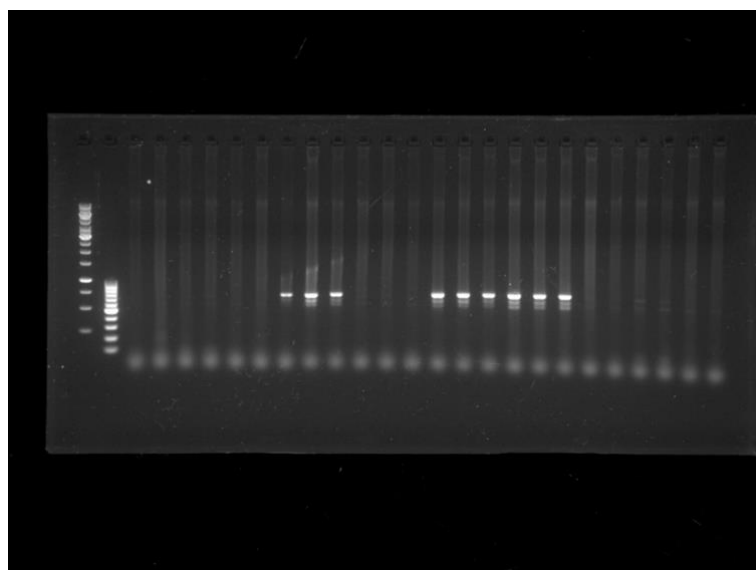

Fig.2c

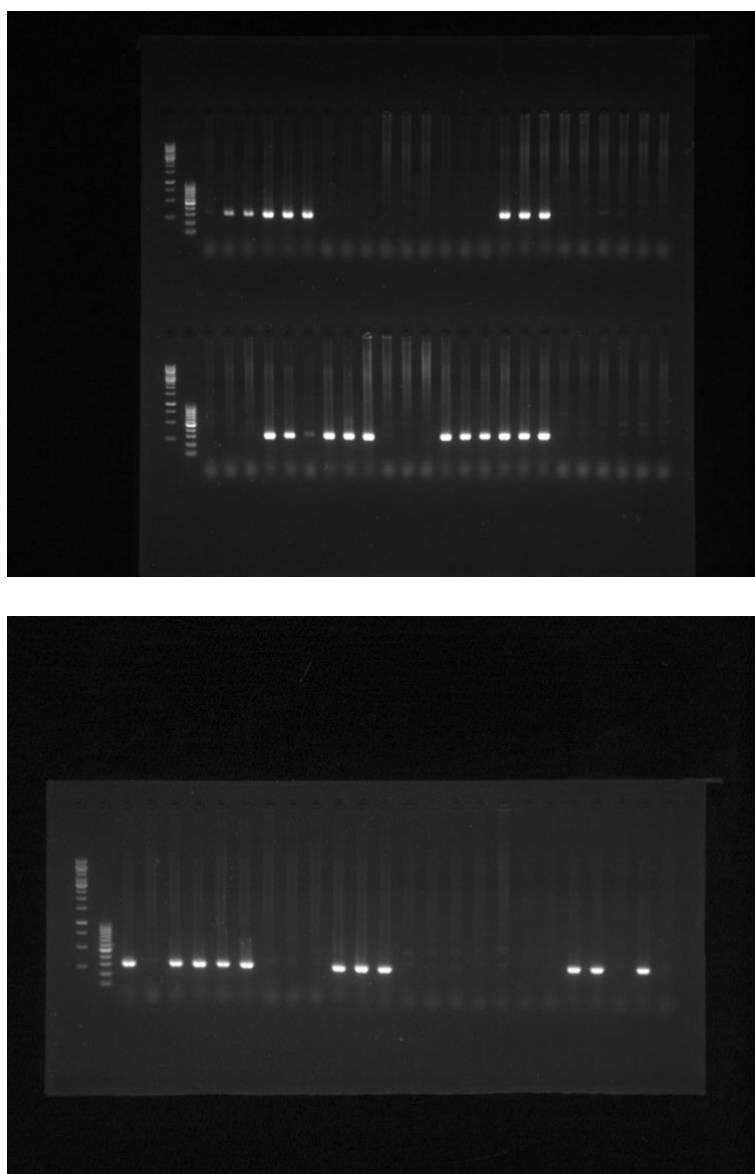

**Fig.2f**

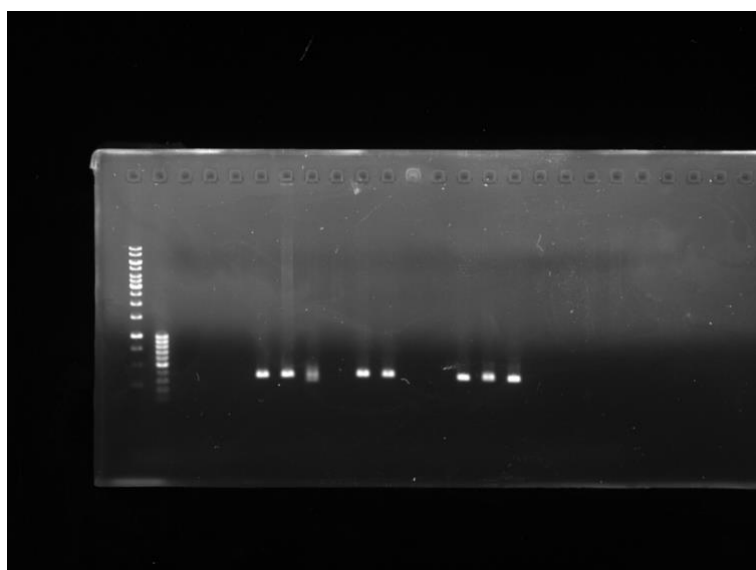

**Fig.3b**

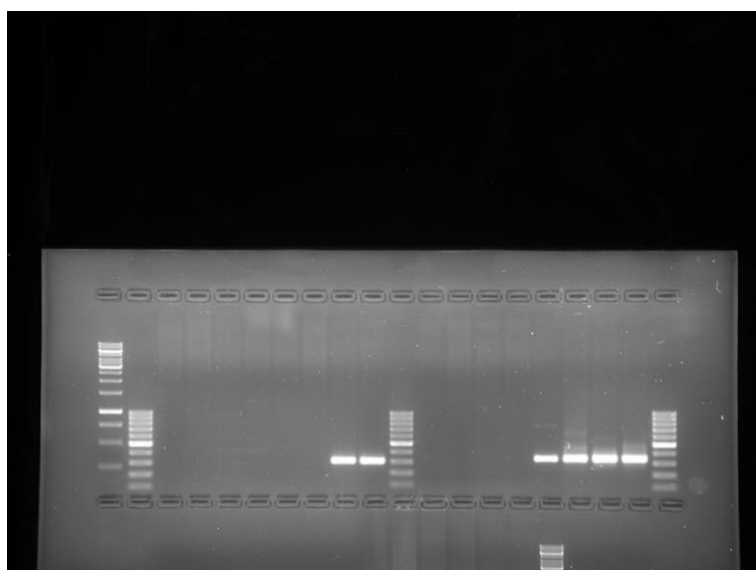

Extended\_Data\_Fig2c

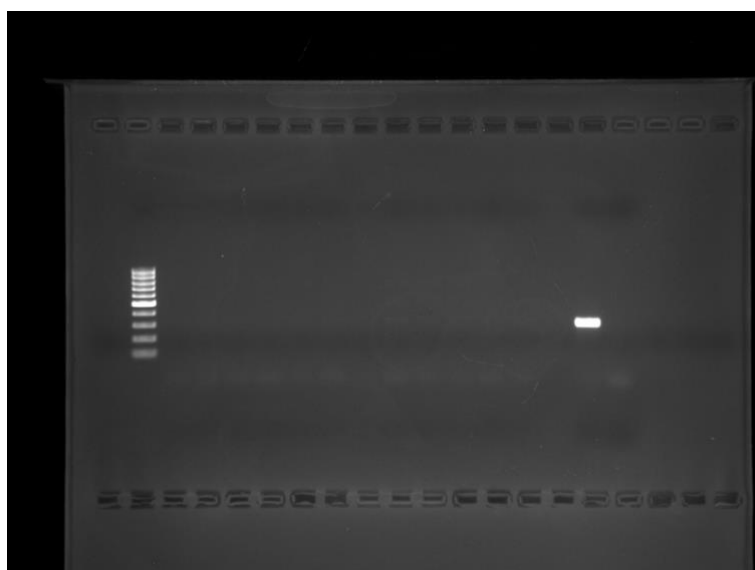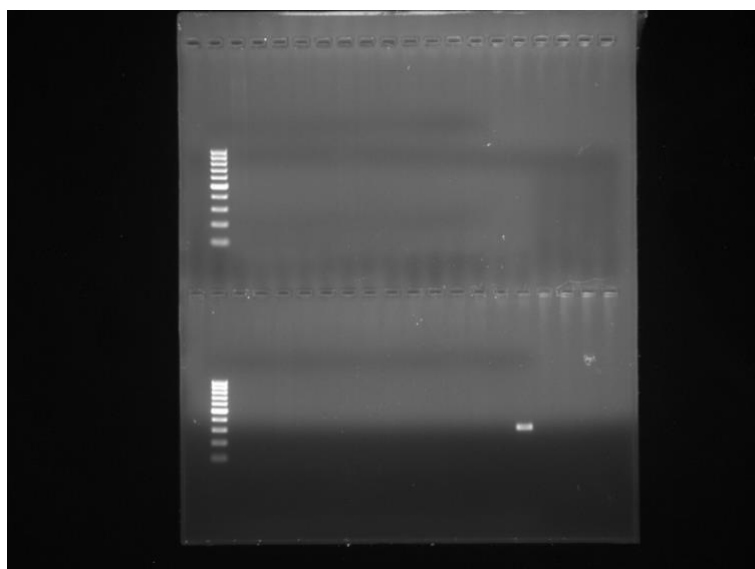

Extended\_Data\_Fig2d

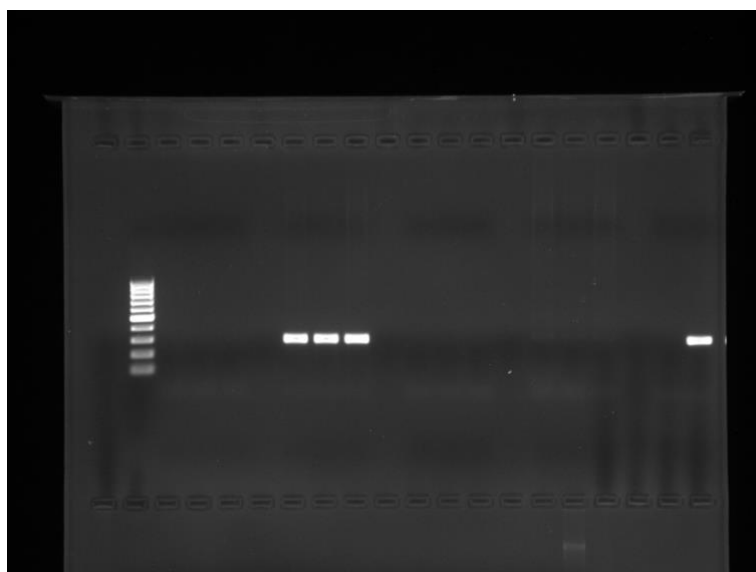

Supplement: Supplementary file 7 — Unprocessed gels. [file 41477_2026_2319_MOESM7_ESM.pdf]
